# The Interleukin Network in Sepsis: From Cytokine Storm to Clinical Applications

**DOI:** 10.3390/diagnostics15222927

**Published:** 2025-11-19

**Authors:** Marcello Candelli, Marta Sacco Fernandez, Gloria Rozzi, Giorgio Sodero, Andrea Piccioni, Giulia Pignataro, Donato Rigante, Francesco Franceschi

**Affiliations:** 1 Department of Emergency, Anesthesiological and Reanimation Sciences, Fondazione Policlinico Universitario A. Gemelli IRCCS, 00168 Rome, Italy; andrea.piccioni@policlinicogemelli.it (A.P.); giulia.pignataro@policlinicogemelli.it (G.P.); francesco.franceschi@policlinicogemelli.it (F.F.); 2 Department of Traslational Medicine and Surgery, Università Cattolica del Sacro Cuore, 00168 Rome, Italy; marta.sacco01@icatt.it (M.S.F.); gloria.rozzi01@icatt.it (G.R.); 3 Pediatric Unit, ASL Brindisi, Perrino Hospital, 72100 Brindisi, Italy; giorgio.sodero01@icatt.it; 4 Pediatric Endocrinology Unit, Perrino Hospital, 72100 Brindisi, Italy; 5 Department of Life Sciences and Public Health, Fondazione Policlinico Universitario A. Gemelli IRCCS, 00168 Rome, Italy; donato.rigante@policlinicogemelli.it; 6 Periodic Fevers Research Center, Università Cattolica Sacro Cuore, 00168 Rome, Italy

**Keywords:** sepsis, IL-1 and sepsis, interleukin 6 signalling, pediatric sepsis, interleukins, cytokines, IL-1, IL-6, IL-10, immune dysregulation, immunotherapy

## Abstract

**Background and Objectives**: Despite major advances in medical science and critical care, sepsis remains a leading cause of morbidity and mortality worldwide: it arises from dysregulated host response to infections and may culminate in organ dysfunction. A hallmark of its pathogenesis is the cytokine storm, in which interleukins (ILs) serve as central mediators of both protective and deleterious immune responses. This review summarizes the current knowledge on the role of ILs in sepsis, emphasizing their potential as biomarkers and therapeutic targets. **Material and Methods**: We analyzed recent clinical and experimental studies focusing on the most studied ILs—including IL-1, IL-6, IL-10, IL-8, IL-12, IL-18, and IL-17—in the pathophysiology of sepsis. Attention was given to mechanistic insights, prognostic significance, and therapeutic strategies targeting IL pathways. **Results**: IL-1 and IL-6 emerged as key pro-inflammatory mediators, amplifying vascular permeability, coagulation activation, and shock, with IL-6 validated as a robust prognostic biomarker. IL-10 was identified as a pivotal anti-inflammatory cytokine, limiting tissue injury but fostering immunosuppression and secondary infections. Other ILs, such as IL-8, IL-12, IL-18, and IL-17, contributed to neutrophil recruitment, Th1/Th17 activation, organ-specific injury, and sepsis susceptibility. Therapeutic interventions targeting ILs, including the IL-1 receptor antagonist anakinra and IL-6 receptor blockade with tocilizumab, have shown promise in selected patient subgroups. **Conclusions**: ILs are central to the immunopathology of sepsis, acting both as drivers of hyperinflammation and mediators of immunosuppression. Their dual role underscores the relevance of ILs as diagnostic and prognostic biomarkers, as well as context-dependent therapeutic targets. Future approaches should prioritize precision immunomodulation aligned with the principles of personalized medicine to improve clinical outcomes in sepsis.

## 1. Introduction

Defined by the Sequential Organ Failure Assessment (SOFA) criteria, sepsis occurs when the host’s dysregulated response to an infection leads to tissue and organ injury, triggering a cascade of events that may have fatal results if not promptly recognized and treated [1]. Sepsis, severe sepsis, and septic shock represent major global healthcare challenges, affecting millions of individuals each year and remaining the leading cause of death in non-cardiac intensive care units [2]. Among these, septic shock is the most critical manifestation, characterized by profound hypotension that reduces tissue perfusion and induces cellular hypoxia [3]. Central to the pathophysiology of sepsis is the intricate interplay within the immune system, where the excessive release of cytokines—commonly referred to as a cytokine storm—represents a fundamental factor [4]. Cytokines are small protein mediators, both pro-inflammatory and anti-inflammatory, secreted by immune and non-immune cells in response to infections: they normally coordinate host defense mechanisms, but if their production becomes dysregulated, they can trigger a profound systemic inflammatory response, leading to endothelial dysfunction, multiorgan failure, and even unfavorable clinical outcomes [5]. Interleukins (ILs) are a group of cytokines produced primarily by white blood cells, which are essential for regulating immune and inflammatory responses. They facilitate communication among immune cells, promote growth, differentiation, and activation, and coordinate defence against infections, inflammation, and other immune challenges. Each IL is designated by a number (e.g., IL-1, IL-2, IL-6), and individual ILs exert distinct functions within the immune system. Some stimulate immune response, whereas others suppress immune responses to maintain homeostasis and prevent excessive inflammation [6]. As central mediators of the immune response, ILs have gained attention as biomarkers for an early diagnosis of sepsis as well as of its prognosis and as attractive potential therapeutic targets. However, their precise clinical utility remains to be fully elucidated. The present review aims to explore the role of ILs in sepsis and to evaluate their potential implications for early recognition of sepsis and septic patients’ management.

### 1.1. Pathophysiology of Sepsis: The Cytokine Storm

Sepsis is a complex syndrome that develops in distinct stages, each reflecting the increasing severity of the host response to infections. It begins with an infection, during which the immune system detects and attempts to eliminate an invading pathogen. However, when this response becomes dysregulated, sepsis can ensue. Clinically, progression is typically classified into three stages: sepsis, severe sepsis, and septic shock, each defined by specific clinical and physiological criteria [7]. The innate and adaptive immune systems are composed of effector cells that secrete cytokines, including ILs, interferons, chemokines, and other signalling molecules. The immune system can recognize and counteract previously unknown pathogens by initiating different defensive pathways. After successful clearance and initiation of healing, it normally returns to a state of homeostasis. These processes are governed by complex mechanisms regulated through multiple activating and inhibitory feedback loops [8]. When this balance is disrupted, however, a cascade of events may be triggered, leading to excessive release of cytokines, referred to as a cytokine storm. This uncontrolled surge amplifies feedback loops, potentially resulting in widespread tissue injury, multi-organ failure, and death [9]. The concept of cytokine storm in sepsis is related to the molecular mechanisms underlying a dysregulated immune response. In particular, the simultaneous activation of multiple cytokine networks contributes not only to hyperinflammation but also to subsequent immune suppression, emphasizing the dual role of ILs in sepsis pathophysiology [10]. Sepsis is defined by an imbalance between pro-inflammatory and anti-inflammatory responses, resulting from an exaggerated and subsequently dysregulated host reaction to a microbial infection. The stronger the pro-inflammatory response, the greater the compensatory anti-inflammatory activity, which may ultimately culminate in significant immunosuppression. The earlier theory of two sequential phases of the immune response—an initial hyperinflammatory phase followed by a “compensatory” anti-inflammatory phase—has largely been abandoned. Instead, the concept of a simultaneous activation of pro- and anti-inflammatory pathways is now most widely accepted [11]. The initial immune response begins when invading microorganisms are recognized by the innate immune system through pathogen recognition receptors (PRRs). These receptors, expressed on immune cells such as dendritic cells and macrophages as well as on epithelial barriers, detect pathogen-associated molecular patterns (PAMPs), which are conserved microbial structures. Examples include lipopolysaccharide (the main virulence factor of Gram-negative bacteria), lipoteichoic acid (a component of the Gram-positive bacterial cell wall), bacterial DNA, flagellin, and peptidoglycan [12]. The interaction between PRRs and PAMPs initiates an intracellular signalling cascade that culminates in the activation of transcriptional programs [13]. Pro-inflammatory mediators such as tumor necrosis factor-α (TNF-α), IL-1β, and IL-6 are markedly elevated and drive the systemic inflammatory response, which, if uncontrolled, contributes to tissue injury, endothelial activation, and multiple organ dysfunction. In parallel, anti-inflammatory counterparts—including IL-10, IL-1 receptor antagonist (IL-1ra), and soluble TNF receptors—are upregulated to counterbalance an excessive inflammation [14,15,16].

### 1.2. Pediatric Sepsis

Pediatric sepsis represents one of the most severe serious bacterial infections (SBIs) in children, with extremely high rates of morbidity and mortality [17,18]. Although it is a potentially fatal condition [19], its clinical presentation is heterogeneous, and distinguishing early sepsis from a severe viral infection—which, while serious, requires a different therapeutic approach—remains challenging [19,20,21]. To optimize the management of pediatric infections, several inflammatory markers, including ILs, are employed as potential early indicators of sepsis, enabling a prompter diagnosis well before microbiological results are available [21,22]. While microbiological confirmation remains the gold standard [17], its diagnostic yield is frequently hampered by prior antibiotic therapy and prolonged turnaround times [19]. Considerable efforts are therefore directed toward identifying reliable early diagnostic markers, and recent evidence indicates that analytical models incorporating IL-1 are among the most effective for an early recognition of SBIs [19,20]. Nevertheless, these biomarkers remain largely experimental, often associated with high costs and limited availability [23]. Consequently, in the routine clinical practice, traditional parameters such as procalcitonin (PCT), C-reactive protein (CRP), or—more recently—presepsin, a soluble fragment of a co-receptor expressed on the membrane of monocytes and macrophages which identifies and binds numerous bacterial products, are more commonly utilized [24].

## 2. Materials and Methods

This narrative review was conducted to summarize current evidence on the role of ILs in the pathophysiology, prognosis, and therapeutic modulation of both adult and pediatric sepsis. A comprehensive literature search was performed in PubMed, Scopus, and Medline up to July 2025. The search strategy combined Medical Subject Headings (MeSH) and free-text terms, including “sepsis,” “interleukins,” “cytokines,” “IL-1,” “IL-6,” “IL-10,” “immune dysregulation,” “interleukin-6 signalling,” and “immunotherapy.” Boolean operators (AND/OR) were applied to optimize sensitivity and specificity. Eligible articles included original studies, systematic reviews, meta-analyses, and clinical trials investigating the role of ILs in sepsis, with a focus on both adult and pediatric populations. Non-English publications were excluded. Background information was drawn from relevant literature published over the past 30 years, whereas the main synthesis was restricted to studies from the last 15 years. Two reviewers independently screened titles and abstracts and assessed potentially relevant articles. Discrepancies were resolved through discussion and consensus. Reference lists of included papers were also screened to identify additional eligible studies. For each study, data were extracted on the IL investigated, study design, population characteristics, clinical outcomes, and therapeutic interventions. Given the heterogeneity of the evidence, a narrative synthesis was performed rather than a quantitative meta-analysis. Findings were thematically organized according to the principal ILs implicated in sepsis (IL-1 family, IL-6, IL-10, IL-8, IL-12, IL-17, and others), their role in immune dysregulation, and their potential as therapeutic targets.

## 3. Key Interleukins Involved in Sepsis

### 3.1. Interleukin-1 Family

#### 3.1.1. Structure, Classification and Function

IL-1, first identified in the 1980s, was the earliest IL to be described: it comprises two principal isoforms, IL-1α and IL-1β, which signal through a common receptor complex (IL-1R1 with IL-1RAcP). Since then, the IL-1 family has expanded to include additional members such as IL-18, IL-33, IL-36, IL-37, and IL-38, for a total of 11 proteins. A naturally occurring receptor antagonist (IL-1Ra) has also been characterized, which competitively binds IL-1R1 without initiating downstream signalling. IL-1 family cytokines are predominantly pro-inflammatory mediators, essential during the early inflammatory response in both adult and pediatric sepsis [25]. They enhance innate immunity by activating natural killer cells, neutrophils, macrophages, and dendritic cells, while also shaping adaptive immunity through the direct stimulation of T cells [25]. Under physiological conditions, these cytokines are crucial for maintaining homeostasis and protecting the host against infections, tissue injury, and environmental stressors [26]. They exert a wide range of direct and indirect effects, including hypothalamic stimulation with subsequent fever, increased vascular permeability, and induction of acute-phase proteins [27].

#### 3.1.2. Individual Roles and Pathophysiological Implications

Among cytochine, IL-38 exhibits predominantly anti-inflammatory properties. By modulating signals downstream of IL-1R1 and IL-36R, it limits NF-κB and MAPK activation. In bacterial infections, particularly sepsis, IL-38 reduces the release of pro-inflammatory cytokines (IL-6, TNF-α, IL-17) and promotes regulatory T cell responses, thereby mitigating tissue injury and improving survival in experimental models [28]. In the context of severe sepsis and septic shock, the excessive release of IL-1, IL-18, and IL-33 leads to cytokine storm characterized by uncontrolled inflammation and widespread tissue damage. Notably, IL-33 shows a positive correlation with PCT levels and severity of sepsis. Monitoring changes in IL-33 is valuable for predicting outcomes and distinguishing sepsis from systemic inflammatory response syndrome (SIRS) [29]. Nevertheless, these cytokines remain essential for mounting an effective immune response during sepsis. IL-36, particularly the IL-36γ isoform, is markedly upregulated in response to bacterial skin infections such as those caused by *Staphylococcus aureus* (*S. aureus*). It promotes neutrophil recruitment and activates other immune cells, thereby contributing to pathogen clearance but also to local inflammation. Excessive or sustained IL-36 activation, however, can lead to tissue injury and chronic inflammatory conditions. As a therapeutic strategy, blockade of the IL-36 receptor with anti-IL-36R monoclonal antibodies has shown promising results. This approach is currently being tested in generalized pustular psoriasis and may prove beneficial for other infectious or inflammatory skin disorders associated with IL-36 dysregulation [30]. IL-18, also known as the interferon-gamma (IFN-γ)-inducing factor, is a pro-inflammatory cytokine with critical immunoregulatory functions [31]. It has been implicated in the pathogenesis and progression of both sepsis and pneumonia, and in non-abdominal sepsis it has been validated as a reliable biomarker and indicator of disease severity [32,33]. Elevated IL-18 levels are associated with multi-organ injury in both viral and bacterial sepsis and correlate with acute kidney injury, overall severity, and mortality [34,35,36]. These findings are consistent with recent evidence showing that circulating IL-18 levels are closely associated with multi-organ injury and unfavorable prognosis in septic patients [37]. Beyond its pathogenic role, IL-18 promotes monocyte-to-macrophage differentiation and migration in patients undergoing orthopedic surgery. In this context, the IL-18/TNF-α ratio has emerged as a robust predictor of clinical severity in sepsis, further underscoring IL-18 as both a biomarker and a potential therapeutic target [38]. Importantly, IL-1 family cytokines have gained recognition as early biomarkers of sepsis, with potential value in identifying patients at increased risk of adverse outcomes.

#### 3.1.3. Therapeutic Strategies

Therapeutic strategies targeting the IL-1 pathway represent a promising approach for sepsis management, with the most available data derived from adult populations and limited evidence in pediatrics [39,40]. A recent clinical study reported that the administration of anakinra in children with secondary hemophagocytic lymphohistiocytosis, multiple organ dysfunction and sepsis was associated with a reduction in inflammatory markers such as CRP over time, although it did not significantly influence overall mortality [41]. Nonetheless, clinical evidence remains scarce, and much of the current knowledge derives from preclinical investigations rather than large-scale clinical trials [42]. The main studies on the IL-1 family in sepsis are summarized in Table 1.

### 3.2. Interleukin-6

This pleiotropic cytokine has a four-helical structure, produced by multiple cell types, including monocytes, macrophages, and endothelial cells, in response to infections and tissue injuries, particularly after exposure to microbial components such as lipopolysaccharide (LPS) [43]. IL-6 signals through two distinct pathways: the membrane-bound IL-6 receptor, known as ‘classic signalling’, and the soluble IL-6 receptor paired with gp130, termed ‘trans-signalling’. Both pathways activate the JAK/STAT3 and MAPK cascades [44,45]. While classic signalling mediates protective and regenerative functions, trans-signalling drives chronic inflammation and contributes to the development of autoimmune disease. In sepsis, IL-6 occupies a central position during both early and late phases of the immune response. Acting as an “alarm signal,” circulating IL-6 levels can rise dramatically- up to a million-fold—thereby amplifying systemic inflammation [46]. IL-6 promotes the acute-phase response by stimulating hepatocytes to produce CRP, serum amyloid-A, and fibrinogen [47,48]. It also induces fever, facilitates leukocyte recruitment, and contributes to vascular endothelial dysfunction, increasing permeability and promoting coagulation, key mechanisms in the development of septic shock and multi-organ failure. During cytokine storm, IL-6 is among the most abundant cytokines detected, often correlating with TNF-α and IL-1β, thereby fuelling hyperinflammation that accelerates vasoplegia, disseminated intravascular coagulation (DIC), and rapid clinical deterioration [49]. Elevated IL-6 has also been associated with organ-specific dysfunctions such as acute kidney injury (AKI), acute respiratory distress syndrome (ARDS), and sepsis-associated myocardial depression [50]. Recent evidence has further substantiated the pivotal role of IL-6 in orchestrating systemic inflammation and highlighted its relevance as a potential therapeutic target. Notably, the dichotomy between classic and trans-signalling pathways of IL-6 not only modulates the trajectory of disease progression but also delineates a critical avenue for targeted interventions in sepsis [51]. IL-6 signals through two main mechanisms: the classic pathway, in which IL-6 binds to the membrane-bound IL-6 receptor (IL-6Rα) on specific target cells, and the trans-signaling pathway, where IL-6 interacts with and binds the soluble IL-6 receptor (sIL-6R), forming a complex that activates gp130 on nearly all cell types [52]. The classic signalling is primarily linked to regenerative, anti-inflammatory, and homeostatic effects, while trans-signalling is associated with pro-inflammatory, endothelial-activating, and tissue-injuring actions. IL-6 trans-signalling disrupts endothelial barrier integrity and increases permeability by sustaining STAT3 activation, leading to the loss of junctional proteins, such as VE-cadherin and ZO-1. It also enhances the production of reactive oxygen species, upregulates ICAM-1, and promotes leukocyte adhesion [53]. Conversely, IL-6 classic signalling supports endothelial survival and reduces apoptosis, inducing only limited levels of inflammatory adhesion molecules. In sepsis, a predominance of trans-signalling can lead to vascular leakage, endothelial activation, tissue edema, and organ dysfunction, whereas classic signalling may help promote protective or resolving immune responses [54]. Selective inhibition of IL-6 trans-signalling, while preserving the classic pathway, thus represents a promising therapeutic strategy to limit vascular and inflammatory damage without suppressing the beneficial functions of IL-6.

These findings highlight IL-6 not only as a biomarker of disease severity but also as a mechanistic driver of organ failure. In pediatric sepsis, IL-6 plays a dual role as both an inflammatory mediator and a biomarker of severity [14]. In early-onset neonatal sepsis, elevated IL-6 levels have been validated as early indicators of SBI, potentially guiding antibiotic initiation [18,19,20,21], even before conventional markers such as CRP become elevated [44]. In neonates at risk of vertical transmission, elevated cord blood IL-6 has shown excellent sensitivity and specificity for early-onset sepsis, though its positive predictive value increases significantly only when combined with other ILs [55]. For example, when IL-6 was assessed together with TNF-α, diagnostic sensitivity markedly improved, rising from 90% and 87.9% for each marker alone to 98.5% in combination [55]. Given these biological functions, IL-6 has been extensively investigated as a prognostic biomarker in sepsis. Numerous studies demonstrate that elevated IL-6 levels correlate with disease severity and mortality. For example, in a prospective cohort of ICU patients with SIRS or sepsis, plasma IL-6 levels on day 3 were strongly predictive of ICU mortality, with concentrations >124.14 pg/mL associated with a six-fold increase in the risk of death [56]. In the emergency departments, IL-6 was an independent predictor of sepsis but did not significantly predict 28-day mortality, in contrast to SOFA score, age, and lactate levels [57]. Beyond its diagnostic and prognostic value, IL-6 has also emerged as a therapeutic target. Tocilizumab, a monoclonal antibody against the IL-6 receptor, has revealed interesting results in both pediatric and experimental sepsis models. In febrile neutropenic children with sepsis, tocilizumab improved mortality when compared to untreated controls [58]. A retrospective pediatric cohort further demonstrated that early administration of tocilizumab within 24 h reduced 28-day mortality from 54.5% to 14.2% and shortened both the duration of septic shock and ICU stay [59]. Table 2 provides a summary of the principal studies investigating the role of IL-6 in sepsis.

### 3.3. Interleukin-10

IL-10 is a central anti-inflammatory and immunosuppressive cytokine, exerting a dual role in sepsis: it limits tissue injury caused by excessive inflammation while simultaneously promoting immunoparalysis during later stages of the disease. It is produced primarily by monocytes, macrophages, and T lymphocytes, and elevated circulating levels are often associated with a worse clinical outcome in septic patients. A recent review described IL-10 as “a pleiotropic cytokine produced by both T cells and macrophages,” capable of suppressing pro-inflammatory cytokine production and antigen presentation, thereby increasing susceptibility to secondary infections in sepsis survivors [60]. In sepsis, intracellular IL-10 production by monocytes and CD4+ T cells is significantly elevated, reflecting a state of systemic immunosuppression. This is accompanied by decreased HLA-DR expression and reduced TNF-α production, consistent with the immune dysfunction characteristic of critical illness [61]. Beyond these cell types, IL-10 can also be secreted by dendritic cells, mast cells, eosinophils, B cells, CD8+ T cells, and regulatory T cells (Tregs). Through this broad cellular origin, IL-10 suppresses MHC class II and co-stimulatory molecules (CD80, CD86), impairs nitric oxide production, and downregulates antigen-presenting capacity [62]. From a vascular perspective, IL-10 contributes to the maintenance of endothelial integrity by attenuating permeability, preserving VE-cadherin expression, a protein critical for cell–cell junctions, and reducing the generation of reactive oxygen species (ROS). Moreover, IL-10 derived from engineered macrophages (IL-10eM) decreases endothelial apoptosis, thereby limiting LPS-induced cell death [63]. In patients with blunt trauma complicated by prehospital hypotension, plasma IL-10 levels were significantly higher during the first seven days post-injury compared with normotensive patients. IL-10, along with other anti-inflammatory cytokines, contributes to the dysregulated inflammatory milieu observed after severe trauma. Its elevation, together with mediators such as IL-6, IL-1β, and IL-17, suggests that the post-traumatic response involves not only excessive pro-inflammatory activation but also inappropriate upregulation of anti-inflammatory pathways. Importantly, increased IL-10 concentrations were associated with worse outcomes, including prolonged mechanical ventilation, higher organ dysfunction scores, and longer ICU or hospital stays [64]. Actually, in the early phase, IL-10 helps protect the host by limiting excessive inflammation and preventing tissue damage. However, when its levels remain elevated over time, it can suppress immune function, leading to immunoparalysis and an increased risk of secondary infections. This prolonged effect ultimately contributes to worse clinical outcomes. An overview of the major studies concerning the IL-10 in sepsis is reported in Table 3.

### 3.4. Interleukin-8

IL-8 is a potent pro-inflammatory chemokine produced primarily by monocytes, macrophages, and endothelial cells: it plays a central role in neutrophil biology as both an activator and chemoattractant, directing polymorphonuclear leukocytes and monocytes/macrophages to sites of infection or injured tissues [65,66]. IL-8 rapidly activates neutrophils by inducing intracellular alkalinization, cytoskeletal rearrangements, and increased glucose uptake, mainly via the sodium-proton exchanger NHE1 and other ion fluxes. These responses are calcium-dependent and rely on ion channels and transporters. Notably, under inflammatory conditions the neutrophil response to IL-8 becomes attenuated, suggesting impaired reactivity during acute inflammation or sepsis [67]. Its effects appear to be concentration-dependent: low IL-8 gradients promote chemotaxis toward inflammatory foci, whereas high concentrations saturating CXCR1/2 receptors induce neutrophil extracellular trap (NET) formation once cells reach the nidus of inflammation [68]. A growing body of evidence supports a close link between IL-8 and NETs. These web-like structures, composed of chromatin, histones, and neutrophil granule proteins, provide rapid antimicrobial defense but can also amplify tissue injury and immunothrombosis when dysregulated [69]. In critically ill septic patients, elevated IL-8 levels have been directly linked to excessive NET generation [70]. Clinically, IL-8 has emerged as a biomarker of sepsis-associated encephalopathy (SAE). In patients presenting with neurological manifestations such as altered consciousness, delirium, or coma, cerebrospinal fluid IL-8 concentrations were elevated, suggesting its potential role in the pathogenesis and in the diagnosis of SAE [71]. Genetic studies further underscore the complexity of IL-8 in sepsis susceptibility. A case–control study in a Chinese cohort demonstrated that the IL-8 -251 A/T polymorphism was associated with a reduced risk of sepsis, particularly among females, elderly individuals, and non-smokers [72]. In contrast, a more recent meta-analysis reported that the IL-8 rs4073 polymorphism increased this susceptibility [73], highlighting the intricate genetic contributions of IL-8 to sepsis pathophysiology. IL-8 has also been implicated in sepsis-induced acute lung injury (ALI), as elevated serum concentrations correlate with disease onset and severity [74]. Importantly, its prognostic value has been demonstrated: in elderly patients with sepsis, IL-8 levels independently predicted mortality in this subset of population. Moreover, in older people, combining IL-8 with the SOFA score significantly improved the diagnostic accuracy of SOFA score alone [75]. Similarly, integration of IL-8 with other biomarkers—including TNF-α, PCT, and heparin-binding protein (HBP)—alongside APACHE II scores enhanced its prognostic performance [76]. Taken together, these findings highlight IL-8 as both a pivotal mediator of neutrophil activation and NET formation, and as a clinically relevant biomarker of sepsis severity, organ dysfunction, and mortality risk [77]. Its multifaceted role positions IL-8 as both a potential therapeutic target and a valuable tool for risk stratification. While anti-IL-8 agents have been approved for other inflammatory conditions such as chronic obstructive pulmonary disease, they are not currently authorized for pediatric sepsis [70].

### 3.5. Interleukin-12

IL-12 is a heterodimeric cytokine composed of two subunits, p40 and p35, which are also shared with other cytokines such as IL-23 and IL-35. IL-12 plays a seminal role in infection control but may also contribute to the pathology of certain diseases, making it a potential therapeutic target. It directly targets B cells and initiates a feed-forward loop involving IL-12 and IFN-γ. This interaction amplifies IFN-γ production and promotes the proliferation and differentiation of plasmablasts from both murine and human B cells. IL-12 and IFN-γ act synergistically on B cells, enhancing each other’s production and activating specific transcriptional programs that drive differentiation into plasmablasts and plasma cells, while limiting germinal centre formation. In vivo, even low concentrations of IL-12 and IFN-γ, ineffective when acting alone, exert potent immunomodulatory effects when combined. Collectively, IL-12 and IFN-γ coordinate extrafollicular responses in both B and T cells, underscoring a fundamental principle of immune regulation with important implications for vaccine development, defence against pathogens, and autoimmunity [78,79]. A major challenge in studying IL-12 arises from the fact that antibodies targeting its subunits can cross-react with other cytokines that share p40 or p35, producing misleading results. To overcome this limitation, a murine anti-IL-12 vaccine was developed, and monoclonal antibodies were engineered to specifically recognize the IL-12 heterodimer without affecting the subunits in related cytokines. One such antibody, MM12A1.6, effectively blocked IFN-γ production and attenuated LPS-induced septic shock following viral infections [80]. Clinically, IL-12 levels have been correlated with infection severity and are significantly higher in children who do not survive infectious episodes [81]. Modulation of dendritic cells, a major source of IL-12, has been proposed as a strategy to improve outcomes in severe infections. However, anti–IL-12 therapies are not currently approved [82].

### 3.6. Interleukin-17 Family

IL-17 is a cytokine family composed of six members (from IL-17A to IL-17F) with diverse roles in the inflammatory cascade during infectious and autoimmune processes [83]. IL-17 is produced primarily by Th17 and Tc17 cells and is critical for the defence of barrier surfaces, such as the skin and mucosae. It binds to a heterodimeric receptor containing an intracellular signalling domain that activates inflammatory pathways, inducing the expression of chemokines, cytokines, and antimicrobial peptides. These mediators recruit neutrophils and enhance protection against extracellular bacteria and fungi. In addition, IL-17 regulates mRNA stability and translation through RNA-binding proteins, establishing a dynamic system of positive and negative feedback. Its biological effects are highly context-dependent: protective during infections and tissue repair, but pathogenic in autoimmune diseases (e.g., psoriasis, rheumatoid arthritis, ankylosing spondylitis), chronic inflammation, and several cancers, where it fosters angiogenesis and even tumor progression [84,85]. In experimental models, IL-17 has demonstrated a protective role during sepsis. In a murine model of severe sepsis, γδ T lymphocytes—particularly the Vγ4 subset—accumulated in the lungs and displayed higher activity than αβ T cells. These cells migrated from the spleen to the lungs in response to chemokine gradients and predominantly produced IL-17, thereby contributing to the host immune response. Depletion of Vγ4 cells reduced IL-17 production and worsened survival, underscoring the protective role of this γδ T cell subset through IL-17 production during sepsis [86]. Further evidence supports a beneficial role for IL-17 in sepsis-associated acute lung injury. In both murine models and patients with sepsis, IL-17D was identified at higher levels in lungs and bronchoalveolar lavage fluid. In LPS-induced acute lung injury, IL-17D levels were significantly reduced in serum and lavage fluid, paralleling findings for septic patients. The administration of recombinant IL-17D increased claudin-18 expression and improved alveolar epithelial barrier integrity, ultimately mitigating lung injury [87]. Key findings from the main studies on the IL-8, IL-12, and IL-17 family in sepsis are outlined in Table 4.

## 4. Cytokine-Mediated Immunosuppression in Pediatric Sepsis

During the immunosuppressive phase of pediatric sepsis, anti-inflammatory cytokines, particularly IL-10, IL-4, and IL-13, play a central role in counterbalancing the initial hyperinflammatory response. Among these, IL-10 is the most potent suppressor. Secreted mainly by monocytes, regulatory T cells, and B cells, it limits immune activation by inhibiting the production of pro-inflammatory mediators such as TNF-α, IL-1, and IL-6, reducing antigen presentation and restraining the activity of macrophages and dendritic cells [88]. IL-4 and IL-13, by contrast, are produced primarily by Th2 lymphocytes and innate lymphoid cells. Acting through the STAT6 pathway, they promote a Th2-oriented immune profile, stimulate B-cell activation, and induce IgE class switching, while simultaneously suppressing Th1-driven responses. These mechanisms are crucial for preventing excessive tissue injury caused by uncontrolled inflammation [89,90]. However, persistent activation of these cytokines during sepsis can shift the balance towards immune paralysis. This state is characterized by impaired pathogen clearance, increased susceptibility to secondary infections, and ultimately worse clinical outcomes [88,89,90]. Elevated circulating levels of IL-10—and, to a lesser extent, IL-4 and IL-13—have been consistently associated with greater disease severity, prolonged hospitalization, and higher mortality in pediatric patients. This duality underscores the critical yet ambivalent role of anti-inflammatory cytokines in shaping the pathophysiology of sepsis during childhood [91].

## 5. Therapeutic Implications

### 5.1. Targeting IL-1 Pathways in Sepsis

Therapeutic strategies targeting ILs in sepsis have yielded mixed yet promising results. Early-phase trials with recombinant human IL-1Ra (anakinra) demonstrated a dose-related survival advantage in patients with sepsis. Recent experimental studies have revealed a paradoxical role of macrophage-secreted IL-1Ra in systemic *Candida albicans* (*C. albicans*) infection. The process occurs in two phases: initially, splenic CD169^+^ macrophages release IL-1Ra into the circulation, hampering early neutrophil recruitment; subsequently, tissue-infiltrating monocyte-derived macrophages produce additional IL-1Ra, further impairing pathogen control. Targeted neutralization of IL-1Ra—through antibodies or genetic approaches—restored neutrophil function, enhanced recruitment to infection sites, reduced maladaptive inflammation, and enabled clearance of *C. albicans*. This intervention protected mice from otherwise lethal sepsis. Type I IFNs were identified as upstream drivers of macrophage IL-1Ra production, aggravating disease severity. Thus, despite its classical anti-inflammatory role, IL-1Ra may paradoxically facilitate fungal dissemination and inflammation, emerging both as a biomarker of candidemia severity and a potential therapeutic target [92,93].

### 5.2. Clinical Applications of IL-1 Inhibition

Long-term blockade of IL-1 has been found to restore the clinical equilibrium in systemic inflammasomopathies of childhood, also if involving the central nervous system [94,95], and IL-1 inhibitors have become cardinal weapons in managing both monogenic innate immunity defects and a plethora of polygenic diseases occurring in children, including Kawasaki disease [96]. Anakinra has also been extensively used for the treatment of macrophage activation syndrome (MAS), an acute life-threatening complication of different rheumatologic conditions occurring in children with a satisfying safety profile and lower discontinuation rates compared to other disease-modifying anti-rheumatic drugs [97]. Clinical subgroup analyses have suggested that IL-1Ra blockade may give benefits to septic adult patients with features of MAS, particularly those with hepatobiliary dysfunction (HBD) and disseminated intravascular coagulation (DIC): in this group, survival improved from ~35.3% with placebo to ~65.4% with anakinra [98]. Moreover, baseline IL-1Ra plasma levels appeared predictive of response: patients with concentrations >2071 pg/mL experienced a significant reduction in adjusted mortality (45.4% vs. 34.3%, *p* = 0.044), whereas no benefit was observed in those with lower levels [99]. A biomarker-guided approach may further refine patient selection. Panels identifying ICU patients with concurrent pyroptosis and ferroptosis signatures—two regulated forms of cell death that drive organ dysfunction and mortality—have stratified patients with the highest risk [58]. Monitoring these pathways could inform precision interventions using IL-1 blockers or ferroptosis inhibitors, thereby improving treatment efficacy [100].

### 5.3. Emerging Cytokine Targets and Alternative Approaches

Other emerging targets include the macrophage apoptosis inhibitor (AIM). Experimental AIM blockade increased survival in sepsis models by reducing systemic inflammation, tissue damage, and bacteraemia, particularly in advanced disease stages. Conversely, recombinant AIM worsened inflammation, increased bacterial spread, and elevated mortality. AIM blockade also reduced IL-10 production by macrophages, neutrophils, and lymphocytes, suggesting that its protective effect may be mediated through suppression of inflammasome activation and modulation of the immune response. These findings position AIM as a potential therapeutic target in sepsis [101]. Furthermore, IL-6 receptor blockade with tocilizumab has also shown encouraging results. In febrile neutropenic children with severe sepsis or septic shock, tocilizumab combined with standard care led to complete recovery in all treated patients (4/4), compared with recovery in only one of three non-treated controls (33%) [58] (Table 5). Promising experimental evidence has shown that curcumin protects cardiomyocytes from sepsis-induced injury by activating the Nrf2/HO-1 pathway, mitigating ferroptosis, and attenuating the IL-6 and IL-1β response to LPS stimulation. These findings underscore the therapeutic potential of natural compounds in modulating oxidative stress and inflammatory damage associated with sepsis [102].

## 6. Future Directions

The role of ILs in sepsis underscores both complexity and heterogeneity of the host immune response. Sepsis should not be regarded as a uniform clinical entity, but rather as a dynamic and multifactorial syndrome characterized by the simultaneous activation of pro-inflammatory and anti-inflammatory pathways. This concurrent immune activity reflects the body’s attempt to eradicate a pathogen while limiting collateral tissue damage. Key ILs such as IL-1 and IL-6 mediate the early hyperinflammatory phase by inducing acute-phase proteins, fever, and immune cell recruitment. Conversely, cytokines such as IL-10 play a central role in dampening excessive inflammation, often leading to immunosuppression. These opposing immune states frequently coexist within the same patient and even within the same tissue compartments, complicating the clinical picture. Such immune duality poses a major therapeutic challenge, as interventions targeting one aspect of the response may inadvertently exacerbate the other, potentially tipping the balance toward further immune dysfunction or uncontrolled infection. A further challenge relies in the marked interindividual variability of sepsis. Age, genetic background, comorbidities, and timing or phase of the septic response strongly influence cytokine profiles and clinical outcomes. Elderly or immunocompromised patients may exhibit attenuated pro-inflammatory responses yet profound immune paralysis, while younger individuals may mount robust cytokine storms. This wide spectrum of immune phenotypes not only drives divergent clinical trajectories but also limits the effectiveness of standardized treatment protocols. It helps to explain why many strategies aimed at neutralizing specific ILs or cytokines have shown promising preclinical results but failed to demonstrate consistent efficacy in large clinical trials. In this context, recent bioinformatic studies have identified several genes involved in IL-18 signalling and cell development as potential diagnostic biomarkers in sepsis, providing additional layers of molecular information that may complement cytokine-based profiling [103]. Precision medicine represents another promising approach. Moving beyond generalized treatment, stratification based on individual immune profiles—such as cytokine expression patterns, biomarker panels, or genetic polymorphisms—may enable clinicians to identify patients most likely to benefit from targeted interventions. For instance, patients with elevated IL-1 activity might respond to anakinra, while those with higher IL-6 activity may benefit from tocilizumab. Incorporating dynamic biomarkers, including serial IL measurements, could further provide insights into disease progression and therapeutic responsiveness. This temporal perspective is crucial, given that the immune profile of septic patients can shift rapidly, necessitating adaptable and timely interventions. Nevertheless, substantial knowledge gaps remain. Translational studies are required to bridge mechanistic insights from preclinical models with clinical applicability, as current experimental systems often fail to fully capture the complexity of human sepsis. Large-scale multi-center clinical trials are urgently needed to rigorously evaluate the safety and efficacy of cytokine-targeted therapies. Adaptive trial designs, biomarker-guided patient selection, and combination approaches that address both pro- and anti-inflammatory components of the immune response will be essential to reflect the evolving and often contradictory nature of sepsis pathophysiology. Only through such a comprehensive and individualized strategy outcome may be improved in this devastating and still insufficiently understood condition.

## 7. Conclusions

ILs are central to the pathophysiology of sepsis, functioning as both drivers and regulators of the immune response. They initiate and amplify early inflammation (e.g., IL-1, IL-6, IL-8), counterbalance it through immunosuppressive signals (e.g., IL-10), and orchestrate downstream processes including immune cell recruitment, vascular permeability, coagulation, and ultimately organ dysfunction. This dual and often paradoxical behaviour underscores the complexity of immune regulation in sepsis. Recent evidence highlights the value of ILs as biomarkers for early diagnosis, risk stratification, and prognosis in septic patients. Elevated levels of IL-6, IL-8, IL-18, and IL-10 are consistently associated with worse outcomes, while temporal trends in cytokine kinetics may provide guidance for therapeutic decision-making. IL-targeted interventions, such as IL-1 and IL-6 blockade, have demonstrated encouraging results in experimental models and in selected patient subgroups, yet clinical translation remains heterogeneous and limited. As our understanding of immune dysregulation in sepsis deepens, it is increasingly clear that a one-size-fits-all approach is inadequate. Inter-individual variability in immune profiles—shaped by factors such as age, comorbidities, pathogen type, and disease timing—demands a precision medicine framework. The future of sepsis management will likely rely on immunophenotyping, real-time cytokine monitoring, and patient stratification to tailor immunomodulatory therapies more effectively. In conclusion, ILs represent not only key immunological mediators, but also valuable clinical biomarkers and potential therapeutic targets. Improving outcomes will require a balanced strategy that addresses both hyperinflammation and immunosuppression. To achieve this goal, sustained translational research coupled with rigorously designed clinical trials will be essential to unlock the full potential of IL-based interventions in sepsis.

## Figures and Tables

**Table 1:** Summary of studies on IL-1 family Cytokines in Sepsis.

Author (Year)	Study Type/ Population	IL-1 Family Focus	Key Findings	Clinical/Therapeutic Implications
Manchikalapati et al., 2024 [25]	Narrative review; pediatric sepsis	IL-1 inhibition	Evaluates clinical utility of IL-1 inhibitors in children with sepsis	IL-1 blockade may be beneficial; need for pediatric-specific trials
Ge et al., 2019 [26]	Review; experimental & clinical data	IL-1 family (IL-1α/β, IL-18, IL-33, IL-36, IL-37, IL-38)	Summarizes biology of IL-1 cytokines in inflammation and sepsis	Highlights IL-1 as central mediator and therapeutic target
Krakauer et al., 2010 [27]	Experimental study; toxic shock, animal models	IL-1 and other pro-inflammatory mediators	IL-1 contributes to cytokine storm and lethality	Supports role of IL-1 blockade in toxic shock/sepsis
Fazeli et al., 2022 [28]	Review	IL-38	IL-38 shows anti-inflammatory properties, reducing IL-6, TNF-α, IL-17	Potential protective role of IL-38 in sepsis and infections
Chang et al., 2015 [29]	Clinical study; septic patients, human	IL-33	Elevated IL-33 correlates with severity and procalcitonin levels	IL-33 may serve as biomarker to predict sepsis outcomes
Buhl et al., 2019 [30]	Review; dermatology/infectious models	IL-36	IL-36γ elevated in bacterial infections, recruits neutrophils	Anti-IL-36R therapy promising; possible sepsis relevance
Roy et al., 2020 [31]	Structural biology, human	IL-18 system	Provides molecular insight into IL-18/IFN-γ axis	Basis for rational drug design targeting IL-18
Herminghaus et al., 2025 [32]	Clinical study; sepsis patients, human	IL-18	IL-18 distinguishes abdominal vs. non-abdominal sepsis	Diagnostic biomarker role
Zheng H., 2025 [33]	Review/clinical correlation	IL-10 and IL-18	IL-18 contributes to severity in pneumonia and sepsis	IL-18 and IL-10 as potential therapeutic target
Qu et al., 2022 [34]	Clinical study; sepsis-induced organ failure, human	IL-18	High IL-18 linked to multi-organ injury and mortality	Prognostic biomarker
Standage et al., 2011 [35]	Review; pediatric sepsis	IL-18 (biomarker focus)	IL-18 among candidate biomarkers for septic shock	Supports pediatric biomarker panels
Sriram et al., 2025 [36]	Clinical cohort; pediatric sepsis with EBV, human	IL-18	EBV seropositivity linked to dysregulated IL-18 and higher mortality	Suggests IL-18 as immune dysregulation marker
Papatheodorou et al., 2025 [38]	Clinical/translational study; post-surgical patients, human	IL-18	IL-18 involved in inflammation resolution; dynamic role	Context-dependent biomarker and therapeutic target
Sen et al., 2016 [39]	Review	IL-1/IL-18 in MAS/macrophage activation	Links IL-1 family to MAS and pediatric hyperinflammation	IL-1 blockade (e.g., anakinra) useful in MAS
Cavalli et al., 2018 [40]	Review	IL-1 blockade	Overview of anakinra across inflammatory diseases	Supports repurposing in sepsis
Rajasekaran et al., 2014 [41]	Case series/clinical experience; critically ill children, human	IL-1Ra (anakinra)	Anakinra improved inflammatory markers in HLH/sepsis/MAS	Suggests therapeutic role, though not definitive
Behzadi et al., 2022 [42]	Review/genetic focus	IL-1 SNPs	SNPs in IL-1 family influence sepsis susceptibility	Genetic variants may guide precision medicine

**Table 2:** Summary of studies on IL-6 in sepsis.

Author (Year)	Study Type/ Population	IL-6 Focus	Key Findings	Clinical/Therapeutic Implications
Tanaka et al., 2016 [43]	Review: Immunity and disease	IL-6 regulation	Describes IL-6’s role in immune regulation and disease pathogenesis	IL-6 is a pleiotropic cytokine; therapeutic targeting explored
Garbers et al., 2018 [44]	Methods in Mol. Biol.; experimental, human	IL-6 classic vs. trans-signalling	Differentiates IL-6 signalling pathways and their biological effects	Trans-signalling inhibition may offer tailored therapy
Eichberger et al., 2022 [45]	Review; neonatal sepsis	IL-6 as diagnostic marker	IL-6 among key markers for early diagnosis of neonatal sepsis	Supports IL-6 as part of biomarker panels
Rose-John S., 2020 [46]	Review	IL-6 signalling	Summarizes IL-6 signalling in health and disease	Provides a rationale for IL-6 targeted therapies
Butterick et al., 2019 [47]	Clinical study; Gulf War Illness, human	IL-6 and CRP	Elevated IL-6 and CRP in affected veterans	IL-6 as marker of chronic inflammation
Roy et al., 2017 [48]	Review; animal model	IL-6 and acute phase proteins	IL-6 regulates acute phase proteins in infection	Highlights IL-6 role across species
Schumertl et al., 2025 [49]	Review; immunopathology	IL-6 signalling and therapy	Summarizes IL-6 biology and therapeutic interventions	Focus on selective IL-6 pathway inhibitors
Song et al., 2019 [50]	Prospective clinical study; septic patients, human	IL-6, PTX3, PCT	IL-6 elevated in sepsis/septic shock, correlates with prognosis	IL-6 useful for diagnosis and definition of prognosis
van Leeuwen et al., 2024 [55]	Systematic review and meta-analysis; neonates	Maternal, cord, neonatal IL-6	IL-6 helpful in early-onset sepsis diagnosis	Confirms IL-6 as a neonatal sepsis biomarker
Pallás Beneyto et al., 2017 [56]	Clinical study, human	IL-6 prognostic value	Confirms IL-6 predictive value for sepsis mortality	IL-6 as a prognostic biomarker in sepsis
Yu et al., 2022 [57]	Clinical study; ED sepsis patients, human	IL-6 diagnostic/prognostic	High IL-6 predicts sepsis and adverse outcomes	IL-6 as an independent predictor of sepsis
Chen et al., 2024 [58]	Retrospective case series; febrile neutropenic children, human	Tocilizumab (anti-IL-6R)	Tocilizumab improved outcomes in severe pediatric sepsis	Suggests therapeutic benefit of IL-6 blockade
Lee et al., 2025 [59]	Clinical study; refractory pediatric septic shock, human	Tocilizumab therapy	Tocilizumab reduced mortality in refractory septic shock	Supports IL-6 blockade as a life-saving intervention

**Table 3:** Summary of studies on IL-10 in sepsis.

Author (Year)	Study Type/ Population	IL-10 Focus	Key Findings	Clinical/Therapeutic Implications
Saxton et al., 2021 [60]	Structural biology study	IL-10 signaling mechanisms	Decoupled pro- and anti-inflammatory functions of IL-10 via structural analysis	Provides rationale for designing IL-10-based immunotherapies
Mazer et al., 2019 [61]	Observational study; septic patients	IL-10 effects on the immune system	IL-10 suppresses innate responses but has variable effects on adaptive immunity	Highlights dual and context-dependent role of IL-10 in sepsis
Vico-Barranco et al., 2021 [62]	Cell line model (TCR signalling)	IL-10 and T-cell signalling	Developed novel T-cell subline for studying IL-10-mediated regulation	Tool for dissecting IL-10 role in T-cell biology
Yi et al., 2022 [63]	In vitro study; engineered macrophages	IL-10 overexpression	IL-10-overexpressing macrophages protected endothelial cells from LPS-induced injury	Suggests therapeutic potential of IL-10-engineered cells in sepsis-related vascular damage
Almahmoud et al., 2015 [64]	Retrospective study; trauma	IL-10 and inflammation dynamics	Prehospital hypotension linked to altered IL-10 levels and worse outcomes	IL-10 as biomarker of dysregulated inflammation in trauma and sepsis

**Table 4:** Summary of studies on IL-8, IL-12, and IL-17.

Interleukin	Reference	Main Findings	Clinical Relevance
IL-8	Matsushima K. et al., 2023 [65]	Historical overview of IL-8 discovery and role as a chemotactic cytokine	Provides background for understanding IL-8’s role in sepsis
IL-8	Matsushima K. et al., 2022 [66]	Comprehensive review on IL-8 biology and evolving functions	Highlights IL-8’s role in inflammatory and infectious diseases
IL-8	Bernhard S. et al., 2021 [67]	IL-8 rapidly alters neutrophil functions, modified under inflammation	Supports role of IL-8 in sepsis pathophysiology
IL-8	Teijeira A. et al., 2021 [68]	Defined IL-8 thresholds for neutrophil chemotaxis vs. NETosis	Suggests IL-8 levels may guide immune dysregulation severity
IL-8	Mao Y. et al., 2023 [71]	CSF IL-8 proposed as biomarker for sepsis-associated encephalopathy	Potential diagnostic marker in pediatric and adult sepsis
IL-8	Fu P. et al., 2019 [72]	IL-8 polymorphism associated with sepsis risk and mortality	Genetic marker for prognosis in sepsis
IL-8	Han T. et al., 2024 [73]	IL-8 polymorphism linked to increased sepsis susceptibility in elderly	Age-specific genetic risk marker for sepsis
IL-8	Liu X.W. et al., 2019 [74]	IL-8 linked with acute lung injury in septic patients	Supports prognostic role of IL-8 in sepsis complications
IL-8	Zhang X. et al., 2024 [75]	Serum IL-8 predicts mortality in elderly sepsis patients	IL-8 as prognostic biomarker in sepsis outcomes
IL-8	Guo S. et al., 2025 [76]	Combined biomarker panel including IL-8 improves sepsis prognosis	Supports use of IL-8 with other markers for risk stratification
IL-12	Elsner R.A. et al., 2024 [78]	IL-12 induces B cell-intrinsic loop promoting extrafollicular responses	Reveals IL-12’s role in adaptive immune regulation
IL-12	Elsner R.A. & Shlomchik M.J., 2025 [79]	IL-12 and IFN-γ coordinate B cell responses	Highlights IL-12’s immunoregulatory function
IL-12	Angurana S.K. et al., 2021 [81]	Measured cytokines including IL-12 in septic children; linked to mortality	IL-12 may be prognostic biomarker in pediatric sepsis
IL-12	Lu J. et al., 2021 [82]	Sepsis impairs dendritic cell progenitors, involving IL-12 dysregulation	Suggests IL-12 role in impaired immune response during sepsis
IL-17	Huangfu L. et al., 2023 [83]	Review of IL-17 family roles in multiple diseases	IL-17 as therapeutic target in inflammatory conditions
IL-17	McGeachy M.J. et al., 2019 [84]	Overview of IL-17 cytokines in health and disease	Contextualizes IL-17 function in sepsis
IL-17	Saran A. et al., 2025 [85]	IL-17 implicated in inflammatory, infectious, malignant disorders	Supports role of IL-17 in systemic inflammation and sepsis
IL-17	de Souza Costa M.F. et al., 2015 [86]	IL-17+ T cells accumulate in lungs, protective role in sepsis	Preclinical evidence of IL-17 protective effects in sepsis
IL-17	Dong S. et al., 2023 [87]	IL-17D protects against LPS-induced acute lung injury	Suggests IL-17D as therapeutic target in sepsis-related ALI

**Table 5:** Effect of IL blockers treatment on survival in septic patients.

Drug	Mechanism of Action	Population	Survival Treated vs. Standard Care (%)	*p*	Reference
Tocilizumab	IL-6 receptor blocker	Febrile neutropenic children	100 vs. 33	0.14	Chen S.H. et al., 2024 [58]
Anakinra	IL-1 Receptor blocker	Septic patients with DIC and hepatobiliary dysfunction	65.4 vs. 35.3	0.007	Shakoory B. et al., 2016 [98]

## Data Availability

No new data were created or analyzed in this study. Data sharing is not applicable to this article.

## Abbreviations

The following abbreviations are used in this manuscript:

IL	Interleukin (followed by the number: IL-1, IL-6, IL-10, IL-8, IL-12, IL-17, IL-18, IL-33, etc.)
IL-1Ra	Interleukin-1 Receptor Antagonist
IL-6R	Interleukin-6 Receptor
TNF-α	Tumor Necrosis Factor-alpha
CRP	C-Reactive Protein
SIRS	Systemic Inflammatory Response Syndrome
SOFA	Sequential Organ Failure Assessment
ICU	Intensive Care Unit
AKI	Acute Kidney Injury
ARDS	Acute Respiratory Distress Syndrome
DIC	Disseminated Intravascular Coagulation
ALI	Acute Lung Injury
SAE	Sepsis-Associated Encephalopathy
PCT	Procalcitonin
HBP	Heparin-Binding Protein
APACHE II	Acute Physiology and Chronic Health Evaluation II
PRR (s)	Pathogen Recognition Receptor (s)
PAMP (s)	Pathogen-Associated Molecular Pattern (s)
LPS	Lipopolysaccharide
NF-κB	Nuclear Factor kappa-light-chain-enhancer of activated B cells
MAPK	Mitogen-Activated Protein Kinase
JAK/STAT3	Janus Kinase/Signal Transducer and Activator of Transcription 3 pathway
NET (s)	Neutrophil Extracellular Trap (s)
SNP (s)	Single Nucleotide Polymorphism (s)
IFN-γ	Interferon gamma
MAS	Macrophage Activation Syndrome
HLH	Hemophagocytic Lymphohistiocytosis
ROS	Reactive Oxygen Species
MHC	Major Histocompatibility Complex
TCR	T-Cell Receptor
Th1/Th2/Th17	T-Helper lymphocyte subsets
Tc17	Cytotoxic T lymphocyte subset producing IL-17
VE-cadherin	Vascular Endothelial cadherin
SBI (s)	Serious Bacterial Infection (s)
eM	Engineered Macrophages (es. IL-10-eM)
AIM	Apoptosis Inhibitor of Macrophages
EBV	Epstein–Barr Virus
gp130	Glycoprotein 130 (IL-6 co-receptor)
HBD	Hepatobiliary Dysfunction
